# Neurological and Neurobehavioral Disorders Associated with *Toxoplasma gondii* Infection in Humans

**DOI:** 10.1155/2021/6634807

**Published:** 2021-10-19

**Authors:** Maxwell A. Virus, Evie G. Ehrhorn, LeeAnna M. Lui, Paul H. Davis

**Affiliations:** Department of Biology, University of Nebraska at Omaha, Omaha, Nebraska, USA

## Abstract

The intracellular parasite *Toxoplasma gondii* is estimated to infect up to 30% of the world population, leading to lifelong chronic infection of the brain and muscle tissue. Although most latent *T. gondii* infections in humans have traditionally been considered asymptomatic, studies in rodents suggest phenotypic neurological changes are possible. Consequently, several studies have examined the link between *T. gondii* infection and diseases such as schizophrenia, epilepsy, depression, bipolar disorder, dysphoria, Alzheimer's disease, Parkinson's disease, and obsessive-compulsive disorder (OCD). To date, there is varying evidence of the relationship of *T. gondii* to these human neurological or neurobehavioral disorders. A thorough review of *T. gondii* literature was conducted to highlight and summarize current findings. We found that schizophrenia was most frequently linked to *T. gondii* infection, while sleep disruption showed no linkage to *T. gondii* infection, and other conditions having mixed support for a link to *T. gondii*. However, infection as a cause of human neurobehavioral disease has yet to be firmly established.

## 1. *Toxoplasma gondii* Infection

*Toxoplasma gondii* is an intracellular protozoan parasite estimated to infect up to one-third of the world population [1]. The parasitic life cycle is complex, involving cats as the definitive host and virtually all other mammals and birds as intermediate hosts [2]. Humans may become infected by eating undercooked meat from an infected host or ingesting oocysts shed in infected cat feces. The oocysts are environmentally resistant and can be infective for lengthy periods [3]. Although uncommon, drinking water has also been a source of infection [4, 5]. Finally, transplacental transmission is a concerning route of infection, transmitted from a mother's primary infection to the developing fetus, often leading to infection-related birth defects [6].

Upon infection, the initial acute stage of infection is effectively controlled in healthy hosts, forcing the parasite to differentiate into a chronic intracellular tissue cyst stage [7]. Intracellular tissue cyst formation takes place in a variety of tissues that include skeletal muscles, the heart, and the brain, with the brain housing a majority of the tissue cyst load in both murine and human models [7–9]. This chronic (or latent) stage of infection, consisting of the bradyzoite parasite stage, is not eliminated via host immune response or even long-term chemotherapeutic options [10].

Although chronic infection has been considered largely asymptomatic in immunocompetent humans, chronic *T. gondii* infection has been shown to produce alterations in rodent behavior [11]. Numerous studies have begun to question whether alteration in behavior is limited to rodents, with the possibility that *T. gondii* infection may affect or instigate behavior or neurological disease state in humans. For example, a recent review by Martinez et al. documented 8 studies relating *T. gondii* to personality changes including aggression, disregard for rules, and self-directed violence [12]. Researchers aptly noted the limitations in measuring human behavior, including complex relationships between the brain, genetics, and social environments; differences in tests used for diagnosing infection and neurobehavioral and neurologic disorders [11]; and difficulty in establishing causal relationships between infection and neurologic disease. Even with the limitations in the studies, the work reinforces the hypothesis that *T. gondii* may have a measurable effect on the human central nervous system.

This present review evaluates current proposed neurobehavioral and neurological disorders associated with *T. gondii* infection and includes schizophrenia [13–15], obsessive-compulsive disorder [16], epilepsy [17, 18], depression [19], bipolar type I disorder [20], dysphoria [21], Alzheimer's [22], and Parkinson's [23]. A review of *T. gondii* literature and its possible relation with neurobehavioral disorders was conducted and organized into Table 1. Where investigated, potential differences between pre- and postnatal infection outcomes are described.

## 2. Mice and Rats as a Host for *Toxoplasma gondii*

Mice and rats are widely used laboratory hosts for *T. gondii* and have been studied extensively as model organisms for infection. Some have gathered evidence that chronic rodent *T. gondii* infection is associated with impaired motor performance, deficits in spatial learning and memory, reduced anxiety, higher activity levels in both novel and familiar environments, sensory attention deficits, altered novelty seeking behavior, and longer reaction times [24–28].

A most interesting reported change in rodents is the reduced avoidance of feline predators and potential attraction to cat urine, with pheromones in the cat urine seeming to generate a signal similar to sexual attraction, thus perpetuating the parasite's sexual lifecycle [11, 29]. The mechanism for this effect is not firmly established, but it is conceivable that nonrandom distribution of and/or activity from the parasite within certain brain regions may contribute to altered behavioral responses. *T. gondii* cysts have been shown to infect up to 92% of brain regions in mice [30], and the selective hypercolonization of various brain regions has been implicated in this modulation of defensive and aversive behaviors in rodents [11, 30, 31]. Some studies suggest that certain brain regions are consistently more infected than others, with tissue cyst density up to twelve times higher [32]. These include the amygdala (responsible for fear responses) and the nucleus accumbens which contains bundles of dopaminergic neurons and is responsible for brain stimulation reward [29, 32, 33]. Areas with consistently low numbers of tissue cysts were reported in the cerebellum, the pontine nuclei, the caudate putamen, the accessory olfactory bulb, and virtually all compact masses of myelinated axons [30, 32, 34, 35]. These studies suggest that nonrandom parasite distribution in the brain may be associated with observed changes within infected animals [36].

Congenital transmission of *T. gondii* in rodents has been considered to have a potential relationship with behavioral disorders, in addition to changes in learning and memory [37–41]. However, definitive findings have not been produced from studies to date. More work is needed in order to link congenital *T*. *gondii* infection in rodent models to cognitive disorders.

## 3. Humans as a Host for *Toxoplasma gondii*

Due to the inability to clear chronic infection once established, easily controlled human pre-/postinfection studies are not ethically feasible, thus making studies of causal relationships less likely. Similarly, it is difficult to establish whether chronically infected adults were infected congenitally or after birth, complicating retrospective studies investigating maternal-fetal transmission effects. *T. gondii* CNS tissue cyst localization in humans is also less well studied, with the data available primarily originating from autopsies of AIDS patients [42]. AIDS patients often have rampant toxoplasmic infections, which cause extensive pathologic lesions to occur throughout the brain. Lesions observed in humans had an apparent stochastic localization, which suggests stochastic localization of the initial parasite tissue cyst [43]. In rodents, tissue cyst localization has been hypothesized to occur more often in the amygdalar brain region due to the modulated fear responses; however, this localization is not apparent in humans [29]. Thus, differences in potential effects between the murine and human host could be due to the overall relatively lower density of parasites in the human brain [44, 45].

## 4. Possible Neurochemistry and Pathway Effects

Many of the neurobehavioral and neurological symptoms that are postulated to be associated with *T. gondii* infection can be correlated to the potential modulation of dopamine in the host brain. *T. gondii* chronic infection is reported to raise whole brain dopamine levels in mice by up to 15% [46]. Hypotheses about the source of the increase in dopamine neurotransmitters include the inflammatory-mediated release of dopamine following cytokine secretion such as interleukin-2 [47] and the existence and activity of tyrosine hydroxylase enzymes in the *T. gondii* genome [48, 49]. It was previously proposed that these tyrosine hydroxylase parasitic genes (AAH1 and AAH2), which encode proteins that produce L-DOPA, interfere with dopamine synaptic transmission and that this interference may lead to neurologic changes [50]. However, a recent AAH2 gene deletion study showed that the enzyme was not required for neurobehavioral changes seen with *T. gondii* murine infection and suggested that effects related to chronic *T. gondii* infection are more likely mediated by neuroinflammation [50]. Variability in immune responses and/or secreted parasite kinases which effect host cell signaling are also potential causes of the observed effects [51]. In addition to potential modulation of dopamine, *T. gondii* infection reportedly affects other neurotransmitter systems such as GABA [52], serotonin [53], noradrenaline [53], nitric oxide [54, 55], kynurenic acid (KYNA) [56], glutamate [57], and the level of proinflammatory cytokines [58]. It is also known that *T. gondii* infection shares transcriptional pathways which overlap other CNS disease states [59]. Taken together, there are a number of possible pathways potentially disrupted by parasite infection which can account for observed differences in model organism and potentially in humans.

Studying *T. gondii* infection and its effects on neurochemistry in humans is more challenging. Yet, human behavioral studies have provided insight to the effects of infection on attributes such as reaction time, masculinity, and personality traits. Humans with *T. gondii* infection appear to have slower reaction times than humans without *T. gondii* infection [60] and also have a higher amount of observed traffic accidents and work accidents, which has been cataloged in four retrospective studies [61–64]. Testosterone levels are higher in college-aged men infected with *T. gondii* [65] and photos of the infected men were rated by females as more masculine [66]. In contrast, female students with *T. gondii* infection had decreased levels of testosterone [65]. Furthermore, men infected with *T. gondii* demonstrated personality traits such as more willingness to disregard rules and to be more suspicious and jealous, whereas infected women tended to be more warm-hearted, outgoing, and easy-going [26]. Both men and women who were infected indicated a decrease in novelty seeking behavior and conscientiousness, but were more entrepreneurial [67–70].

Although infection with *T. gondii* was previously considered asymptomatic, there are clear suggestions of phenotypic CNS alterations due to chronic *T. gondii* infection. To study how far-reaching these effects could be, research continues to attempt to correlate *T. gondii* infection with changes in behavior and manifestations of neurobehavioral or neurological disorders. Table 1 lists published studies evaluating the CNS disorder associations.

## 5. Detecting *Toxoplasma gondii*

The diagnosis of *T. gondii* infection has traditionally been made by performing serological tests for the presence of antibodies produced by the host immune system in response to an exposure. Cerebrospinal fluid (CSF) can also be drawn and used to detect the *T. gondii* antibodies although this technique is more difficult and expensive. Most studies listed in Table 1 used an enzyme-linked immunosorbent assay (ELISA) and seropositivity of *T. gondii-*IgG antibodies to detect whether subjects had been exposed to the parasite. Serointensity, or the relative concentration of IgG antibodies present in serological readings, was also measured in some studies, although the importance of differences in serointensity or the causes of these differences have not been established. Notably, these methods do not permit the ability to distinguish maternal transmission from postnatal acquisition if evaluated several weeks or more after birth.

Overall, the categorized studies reviewed in Table 1 suggest potential correlations between *T. gondii* infection and some neurobehavioral or neurologic disorders. Each of the evaluated disease categories that were evaluated are discussed below.

## 6. Methodology and Results

The methodology utilized to find research articles to document current potential neurobehavioral disorders associated with chronic infection of *T. gondii* included utilizing specific terms in PubMed. These terms include “*T. gondii* neurobehavioral disorders,” “*T. gondii* neurologic disorders,” and “Effects of chronic toxoplasmosis” limited to the year 1990 to present. Cited sources from these papers were also used as resources for finding related studies. If studies included differentiation between maternal-fetal transmission and postfetal acquisition, these were noted.

### 6.1. Alzheimer's Disease

Kusbeci et al. found that *T. gondii* seropositivity was significantly higher in 34 Alzheimer's patients compared to 37 control patients (*p* = 0.005) [22]; however, the Alzheimer's population was substantially older (68.1 vs. 62.9 years). Prandota suggests that Alzheimer's may be associated with congenital transmission; however, there was no significant correlation between them [71]. More recent studies with larger populations did not detect correlations or statistical significance between Alzheimer's and *T. gondii* infection [72–78]. Thus, it is undetermined if Alzheimer's disease and *T. gondii* infection are linked. Notably, however, *T. gondii* exposure may be associated with cognitive decline in older persons [79, 80]. Studies in murine models have shown an inconsistency between the linkages between Alzheimer disease progression and *T. gondii*. Both Torres et al. and Mahmoudvand et al. found that *T. gondii* infection induced or worsened pathological progression and signs associated with Alzheimer's disease [28, 75]. However, Jung et al. suggested otherwise, stating that *T. gondii* infection suppressed the neurodegenerative-associated pathogenesis in an Alzheimer's disease murine model [81]. Therefore, future studies must be done to determine whether an association between *T. gondii* infection and cognition changes due to Alzheimer's disease exists.

### 6.2. Bipolar Disorder

It has been contemplated that latent *T. gondii* may trigger CNS oxidative stress, leading to immune-inflammatory processes that could promote or resemble CNS changes seen in bipolar disorder [82–84]. In studies conducted by Pearce et al. and Hamdani et al, it was found that patients seropositive for *T. gondii* infection were approximately 2.3- and 2.7-folds more likely to have a history of bipolar disorder type I with manic and depressive symptoms than respondents who tested negative for the *T. gondii* antibody [85, 86]. A more recent 2019 study also found a possible linkage between depressive bipolar disorder and *T. gondii* seropositivity (*p* = 0.04) [87]. While Stich et al. found no significant association between bipolar disorder and *T. gondii*, a multitude of studies suggest otherwise [88–92]. Further, studies of maternal-specific transmission of *T. gondii* infection have not found significant association with bipolar disorder [91, 93–95].

The CNS-acting drugs haloperidol, cyamemazine, loxapine, zuclopenthixol, and fluphenazine (but not valproate) showed at least moderate antitoxoplasmic activity when evaluated *in vitro* [96, 97]. *T. gondii*-seropositive patients with bipolar disorder I who were treated with valproate, haloperidol, zuclopenthixol, cyamemazine, and/or loxapine experienced on average two fewer lifetime depressive episodes than those who were treated with medications with lower *in vitro* anti-*T. gondii* activity [98, 99]. However, none of these drugs have been shown to affect the latent tissue cyst stage of the parasite. While it is conceivable that *T. gondii*-positive patients may derive enhanced benefit from receiving medications that have shown anti-*T. gondii* activity, it is notable that standard antiparasitic treatment options have not shown marked improvement in patient psychopathology [100]. Taken together, the available studies evaluating *T. gondii* infection associated with some form of bipolar disorder (Table 1) suggests a linkage more often than not.

### 6.3. Depression, Dysphoria, and Hopelessness

Studies have been inconsistent in linking *T. gondii* infection with depression, with a majority of studies finding that *T. gondii* and depression are not linked. Alvarado-Esquivel et al. suggested a linkage between depression and *T. gondii*, especially in younger populations ages 17-30 years old [101], but this study was limited in geography. Duffy et al. also found a potential correlation between chronic *T. gondii* infection and depression in women veterans indicating a higher depression score [102]. Wadhawan et al. found that the relationship between *T. gondii* IgG seropositivity and ethnicity was statistically significant with regard to the Hispanic population. This could point to a previously unknown relationship between *T. gondii* and those of Hispanic origin [21]. In a study presented by Yalın Sapmaz et al., there was also a suggested relationship between young adolescents, depression, and *T. gondii* seropositivity [103]. This association is also seen in a few other studies as well [104, 105]. However, in a study conducted by Mahmoud et al., depression was correlated to reactivated *T. gondii* and not chronic infection of *T. gondii* in BALB/c mice [106]. In a study conducted in 2020, a similar result was found where chronic infection of *T. gondii* did not appear to have correlation with depression, yet there was a correlation with the acute stage [107]. Multiple other studies found no association between latent *T*. *gondii* and depression [74, 108–114]. Flegr and Hodný postulate that a pathogen responsible for mood disorder may not be *T*. *gondii* but rather a different feline-borne pathogen [110]. Presently, a link between depression and *T. gondii* infection cannot be determined until further studies are completed.

### 6.4. Epilepsy

Tachyzoite infection of neurons has shown to deregulate calcium influx upon stimulation with glutamate [115], and calcium is known to play an important role in the initiation and spread of seizure activity. Two meta-analysis studies suggest a positive correlation between *T. gondii* infection and development of epilepsy [17, 18], whereas other studies evaluated the potential relationship between epilepsy and *T. gondii* but found no statistical significance [74, 116–118]. Stommel et al. found a possible association with cryptogenic epilepsy and chronic *T. gondii* infection [119]. In addition, it was reported that rats with chronic and acute *T. gondii* infection had a lower seizure threshold compared to uninfected healthy rats, and the average seizure threshold was restored only once a pharmacological blockade of dopaminergic receptors occurred [120]. Given the results of these studies, it is conceivable that the effects of chronic *T. gondii* infection on dopamine concentrations and/or acute *T. gondii* infection on calcium concentrations may increase the risk of seizures and epilepsy.

Infection by *T. gondii* has also been associated with changes in sex hormone levels, such as increased testosterone serum concentrations [121–123]. Testosterone concentration is known to affect dopamine signaling pathways, changing the sensitivity of the nigrostriatal pathway to dopamine [124]. This may suggest that *T. gondii* may be correlated to specific types of epilepsy, such as hormone-driven epilepsy [125]. One hormone-related epilepsy, catamenial epilepsy, may be influenced by *T. gondii* infection due to modified dopamine levels, which are linked to the level of estrogen [126]. There is a reported strong correlation (*p* < 0.001) between the prevalence of epilepsy and seropositivity of toxoplasmosis in pregnant women [18]. Further, a case study reports a patient with congenital toxoplasmosis and temporal lobe epilepsy, suggesting a relationship between the two [127]. Altogether, multiple studies point towards the idea that epilepsy is linked with *T. gondii* seropositivity [114, 128–132], while others find no link. Perhaps certain types of epilepsy may be more associated with *T. gondii* infection.

### 6.5. Headaches and Migraine

Chronic *T. gondii* infection alters inflammatory cytokines, immunologic responses, and other biochemical responses such as the downregulation of nitric oxide, some of which are posited to be a contributing factor to headaches [133–135]. A study of recurrent headaches in pediatric neurology patients found that 11% were positive for chronic *T. gondii* infection, and the majority of these patients had headaches most often in the frontal region [135]. A more recent study found that recurrent headaches were only statistically correlated with *T. gondii* on the basis of serointensity but were not correlated with seropositivity [136]. An earlier study found statistical significance between chronic *T. gondii* infection and migraine, describing 44% of patients experiencing migraine being positive for chronic *T. gondii* infection versus only 26% in healthy control subjects [134]. Multiple other studies support this association between *T. gondii* infection and migraines [137, 138]. However, while headaches and lymphadenopathy are the most commonly reported symptoms of acute toxoplasmosis, the relationship between recurrent headaches and migraine with chronic *T. gondii* infection is still uncertain [134].

### 6.6. Obsessive-Compulsive Disorder (OCD)

Obsessive-compulsive disorder is thought to involve alterations in dopaminergic and serotonergic pathways [139]. In one study, the prevalence for anti-*T. gondii* IgG antibodies among OCD patients (48%) was found to be significantly higher than the prevalence in healthy volunteers (19%) [16]. Another study found a 2.5-fold increase of OCD prevalence among 7471 subjects with *T. gondii* chronic infection [140]. In a study conducted by Akaltun et al., there was a significant relationship between serum *T. gondii* IgG positivity and increased risk of OCD (4.9 fold) for children and adolescents [141], but a similar study of pediatric OCD found no statistical difference in seropositivity [142]. From a meta-analysis conducted by Chegeni, it was determined that 26% of patients with OCD were positive for chronic infection compared to those without OCD at 17% [143]. However, they described the limitations of the meta-analysis failing to fully represent the general population due to the samples being recruited through Facebook [143]. Other cross-section studies found no association between OCD and *T. gondii* infection [74, 111, 137]. Ultimately, the findings cumulatively suggest that there may be an association between OCD and chronic *T. gondii* infection in children, adolescents, and adults, but more studies must be done to confirm it.

### 6.7. Sleep

*T. gondii* infection reportedly affects neurotransmitter systems such as dopamine [48], GABA [52], serotonin [53], and noradrenaline. These neurotransmitters are involved in the sleep/wake cycle, and alterations in neurotransmitter concentrations or the sleep/wake cycle could lead to complications including psychiatric conditions [144], behavioral problems [145–147], car accidents [148], suicide, and cognitive deficits [149]. Sleep disturbances and disorders have long been known to affect mentality and are linked with depression [150]. In a study performed by Ahmad et al. attempting to link *T. gondii* infection and sleep disturbance, such as quality or quantity of sleep, no linkage was found [151]. There also was no association found between *T. gondii* and sleepiness or sleep apnea in obese patients [152]. Indeed, in a study conducted by Corona et al., *T. gondii* seropositivity was linked to less sleep problems [153]. Taken together, there is no significant evidence of *T. gondii* associated with sleep disturbances.

### 6.8. Suicide

In 2016, nearly 45,000 Americans aged 10 or older died by suicide (Center for Disease Control and Prevention). Suicide is an increasing risk in the United States, especially for those mentally ill. The studies outlined in Table 1 suggest a relationship between suicide and *T. gondii* infection, as similarly reviewed by others recently [49, 154–160]. In a study by Okusaga et al., significance was found between *T. gondii* infection and suicide attempts in the subgroup of patients younger than 38 years old [49].Yagmur et al. also found significance between suicide attempts and *T. gondii* infection in a population of infected individuals in the age group 24 ± 7.6, whereas many of the other studies used populations of older individuals [154, 155]. Thus, the age of infection could be a contributing factor in the risk of individuals infected with *T. gondii* and suicide. Bak et al. also found *T*. *gondii*-seropositive patients to have a higher Hamilton depression rating score than seronegative suicide attempters [156]. While Alvarado-Esquivel et al. did find a possible association between high anti-*T. gondii* antibody levels and suicide attempts, Arling et al. found no significance between seropositivity and suicide [161, 162]. Continued studies, particularly those correlated with patient age, may show more association with suicide and *T. gondii* infection.

### 6.9. Parkinson's Disease

With *T. gondii*'s effect on dopamine neurotransmitter concentration, it has been proposed that *T. gondii* may play a role in the etiology of neurological diseases such as Parkinson's [20]. In Parkinson's disease (PD), dopamine levels are decreased and dopamine producing neurons are destroyed [81]. Vlayjinac et al. researched Parkinson's disease with its relationship to viral and bacterial infections and found that it was significantly related to mumps, scarlet fever, influenza, herpes simplex, and whooping cough infections but not related to measles, chicken pox, and tuberculosis. Miman et al. suggested that the inflammation and degeneration of dopamine-producing neurons caused by *T. gondii* could cause PD [23]. They found a significantly different prevalence for *T. gondii* antibodies in Parkinson's patients versus controls, 42% and 23%, respectively. Furthermore, Mahami-Oskouei et al. discovered a statistically significant association between Parkinson's disease and owning a cat (*p* = 0.03) although the *T. gondii* status of owned cats was not established. However, Mahami-Oskouei et al. found no significant association between IgG-positive titers and Parkinson's disease [163], and the meta-analysis conducted by Zhou et al. concluded that there is no linkage between PD risk and *T. gondii* infection [164]. While multiple studies suggest no significant linkage between PD and *T. gondii* infection [48, 165, 166], Ramezani et al. found higher anti-*Toxoplasma* IgG antibodies in patients with idiopathic PD. More studies may be valuable to examine the potential relationship between Parkinson's disease and *T. gondii*, as well as other infections.

### 6.10. Schizophrenia

The connection between schizophrenia and *T. gondii* infection has been studied since at least 1956 [167]. Patients with schizophrenia tend to have abnormal dopamine neurotransmitter levels as well as an abnormal glutamate and gamma aminobutyric acid (GABA) level [168]. Dopamine imbalance driven by the parasite may contribute to disease progression or intensity. The imbalance of the dopaminergic, mesolimbic, and mesocortical pathways, which are responsible for motivation, emotional responses, and reward, is also implicated in schizophrenia [169].

The immune system could also play an effect in the possible relation between *T. gondii* and schizophrenia [170]. Adaptive immune CD8^+^ T cells are important in controlling *T. gondii* infection and interact with MHC Class I alleles on infected cells. These CD8^+^ T cell responses may be relatively decreased following infection with some virulent strains of the parasite, such as the nontissue cyst generating RH strain [171, 172]. Downregulation of CD8^+^ T cell responses has been recognized as a commonality in individuals with schizophrenia [173]. The downregulation of CD8^+^ T cell responses caused by chronic *T. gondii* infection may be associated with the downregulation seen in individuals with schizophrenia. It should be noted that the MHC class I allele differs between hosts, which has been shown to affect the susceptibility to infection and disease significantly [174, 175].

In a meta-analysis of 38 studies, Torrey and Yolken determined the presence of *T. gondii* antibodies in patients to be an intermediate risk factor for schizophrenia [176]. Cetinkaya et al. determined that there were increased levels of serum anti-*T*. *gondii* IgG in patients with schizophrenia, while Leweke et al. found elevated anti-*T*. *gondii* antibodies in both serum and cerebrospinal fluid in patients with recent-onset schizophrenia [14, 15]. More recently, a study of 81,912 individuals from the Danish Blood Donor Study also found evidence that *T. gondii* and schizophrenia were statistically associated [177] while Muflikhah et al. also showed that seropositivity was higher at 69% for the group of patients with schizophrenia compared to the control group at 66%; however, the latter was not considered statistically significant [178]. In addition, Alipour et al. and Kezai et al. showed a significant difference of the seropositivity rate between controls and patients with schizophrenia: specifically, 67.7% in patients with schizophrenia vs. 37.1% in control patients and 70% in patients with schizophrenia vs 52.9% in controls [179, 180]. Significance has been further analyzed by the study done by Fond et al. finding that *T*. *gondii* is three times more frequent in the schizophrenia population than that in general populations [181]. Despite these possible associations, countries with a higher prevalence of *T. gondii* seropositivity have not shown a corresponding increased schizophrenia prevalence [182].

One component of these investigations is the association of *T. gondii* positivity, schizophrenia, and gender, but findings are inconsistent. For instance, Karabulut et al. showed no significant difference between *T. gondii* positivity and schizophrenia in respect to gender and age [183]. However, other studies have found a higher prevalence of *T. gondii* positivity in schizophrenic males compared to females [184, 185]. Yet, Khademvatan et al. showed the opposite effect, finding a significant increase of seropositive schizophrenic females compared to seropositive schizophrenic males [186]. Ultimately, the linkage to gender must be further studied because a conclusive finding can be made. In regard to severity of symptoms, Esshili et al. notes that men with schizophrenia tend to have more severe negative and cognitive symptoms; additionally, patients have higher age of disease onset and an overall less favorable course [187].

Possible associations have also been found between maternal transmission of *T. gondii* infection and schizophrenia development in individuals. In a study conducted by Brown et al., subjects with schizophrenia spectrum disorders were 2.6-folds more likely to have mothers with detectable *T. gondii* IgG antibody titers [188]. In both prenatal and postnatal toxoplasmosis, there were linked findings for the risk factor of schizophrenia due to increase levels of homovanillic acid and dopamine which can be implicated with schizophrenia pathogenesis. Brown's 2011 review supports similar findings that prenatal infection of *T. gondii* may be a potential risk factor for schizophrenia [189]. Brown also suggests that differential consequences on fetal brain formation and development of schizophrenia may be due to the unique mechanisms of prenatal infection. Multiple other studies have related conclusions distinguishing the association between maternal prevalence and the increased risk of schizophrenia [190–194], including a suggestion that schizophrenia is more likely to develop with congenital toxoplasmosis than acquired toxoplasmosis based on behavioral testing [162]. More studies are needed to confirm this linkage.

Overall, the potential relationship with schizophrenia is the longest studied neurobehavioral disorder with regard to *T. gondii* infection, and the results taken together indicate a strong possible association between *T. gondii* infection and schizophrenia [195].

## 7. Conclusion

Chronic infection by *T. gondii* is considered lifelong in all hosts, including humans. Despite the number of medicinal therapies available to combat acute infection, evidence does not exist for a treatment consistently capable of clearing the tissue cyst stage in chronic infection, although new drug discovery is ongoing [10, 196, 197]. Thus, it is difficult to establish the causality of potential diseases reviewed here in humans. Furthermore, experimentally increasing *T. gondii* levels in mice or other models have been insufficiently studied to provide dose-dependent observations.

*T. gondii* seropositivity has been shown to differ significantly between countries, diverse topographical regions within the same country, and individuals of different ethnicities that live in similar regions [21, 198–204]. In considering linkages to *T. gondii* human infection, differences in lifestyle, cooccurring substance use disorders, varying differences in host species and/or strain, mode of infection (tissue cysts or oocysts), and/or timing of infection (in utero, childhood, or adulthood) may affect the findings of each study [7, 21, 205].

In conclusion, available studies on schizophrenia most consistently support a correlation of *T. gondii* and these disorders, while the literature on other disorders is mixed. Therefore, the relationship between *T. gondii* infection and neurological or neurobehavioral disorders in humans should continue to be studied.

## Figures and Tables

**Table 1:** Studies conducted examining possible linkages of *T. gondii* infection with neurobehavioral symptoms and disorders.

Disease	Reference	# of subjects	Mean age (yr)	Linkage	Finding
Alzheimer's	Kusbeci et al. [22]	71	68 ± 16	+	*T. gondii* seropositivity seen to be associated with Alzheimer's
	Mahami-Oskouei et al. [76]	150	76 ± 7.2	-	*T. gondii* seropositivity not seen to be associated with Alzheimer's
	Perry et al. [72]	219	80 ± 7.2	-	*T. gondii* seropositivity not seen to be associated with Alzheimer's
	Bouscaren et al. [73]	1662	73 ± 7.0	-	*T. gondii* seropositivity not seen to be associated with Alzheimer's
	Cong et al. [74]	88	16 − 91	-	*T. gondii* seropositivity not seen to be associated with Alzheimer's
	Menati Rashno et al. [77]	87	62 ± 21	-	*T. gondii* seropositivity not seen to be associated with Alzheimer's
	Menati Rashno et al. [77]	174	21 ± 69	-	*T. gondii* seropositivity not seen to be associated with Alzheimer's

Bipolar disorder	Pearce et al. [85]	7440 (M)	15 − 39	+	*T. gondii* seropositivity seen to be associated with bipolar disorder I
	Hamdani et al. [86]	216	47 ± 11	+	*T. gondii* seropositivity significantly different between groups (3.6x increased likelihood)
	Fond et al. [99]	266	44 ± 13	+	*T. gondii* seropositivity seen to be associated with more bipolar disorder depressive episodes
	Del Grande et al. [83]	101	≥40	+	*T. gondii* seropositivity seen to be associated with bipolar disorder
	Alvarado-Esquivel et al. [87]	462	40 ± 14	+	*T. gondii* seropositivity seen to be associated with a specific type of bipolar disorder
	Afifi et al. [82]	40	32 ± 8	+	*T. gondii* seropositivity and serointensity seen to be associated with bipolar disorder and oxidative stress
	Hamdani et al. [84]	334	18 − 65	+	*T. gondii* seropositivity seen to be associated with bipolar disorder
	Frye et al. [88]	52	–	+	*T. gondii* seropositivity seen to be associated with bipolar disorder
	Hamdani et al. [89]	78	42	+	*T. gondii* seropositivity seen to be associated with bipolar disorder
	Oliveira et al. [90]	305	41 ± 14	+	*T. gondii* seropositivity seen to be associated with bipolar disorder
	Stich et al. [92]	46	–	-	*T. gondii* seropositivity not seen to be associated with bipolar disorder
	Del Grande et al. [95]	7440	15 − 39	-	*T. gondii* seropositivity not seen to be associated with bipolar disorder
	Chaudhury and Ramana [93]	216	–	+	*T. gondii* maternal seropositivity seen to be associated with bipolar disorder in offspring
	Mortensen et al. [91]	127	27	-	*T. gondii* maternal seropositivity not seen to be associated with bipolar disorder in offspring
	Freedman et al. [94]	214	Maternal: 27.7 Paternal: 32.2	-	*T. gondii* maternal seropositivity not seen to be associated with bipolar disorder in offspring

Depression/dysphoria/hopelessness	Alvarado-Esquivel et al. [101]	445	38 ± 13	+	*T. gondii* seropositivity seen to be associated with depression
	Duffy et al. [102]	70	47 ± 10	+	*T. gondii* seropositivity seen to be associated with depression and dysphoria
	Yalin et al. [103]	37	11 − 18	+	*T. gondii* seropositivity seen to be associated with depression
	Nasirpour et al. [103]	174	62 ± 22	+	*T. gondii* seropositivity seen to be associated with depression
	Groër et al. [105]	414	–	+	*T. gondii* seropositivity seen to be associated with depression
	Alvarado-Esquivel et al. [109]	400	23 ± 18	-	*T. gondii* seropositivity not seen to be associated with depression in pregnant women
	Gale et al. [108]	1846	29 ± 0.4	-	*T. gondii* seropositivity not seen to be associated with major depressive disorder
	Flegr and Hodný [110]	5535	32 ± 13	-	*T. gondii* seropositivity not seen to be associated with depression
	Wadhawan et al. [21]	306	46 ± 16	-	*T. gondii* seropositivity not seen to be associated with dysphoria and hopelessness
	Pearce et al. [20]	7440 (M)	15 − 39	-	*T. gondii* seropositivity not seen to be associated with unipolar mood disorders such as depression
	Cong et al. [74]	78	16 − 91	-	*T. gondii* seropositivity not seen to be associated with depression
	Zaki et al. [111]	168	35 ± 9	-	*T. gondii* seropositivity not seen to be associated with depression
	Nourollahpour Shiadeh et al. [112]	360	28 ± 5	-	*T. gondii* seropositivity not seen to be associated with depression
	Shahnaz et al. [186]	180	–	-	*T. gondii* seropositivity not seen to be associated with depression
	Abd El-Aal et al. [114]	178	36 ± 14	-	*T. gondii* seropositivity not seen to be associated with depression

Epilepsy	Ngoungou et al. [17]	2888 (M)	All ages	+	*T. gondii* seropositivity seen to be an epilepsy risk factor
	Palmer [18]	204 (M)	No ages listed	+	*T. gondii* seropositivity seen to be associated with epilepsy, especially cryptogenic epilepsy
	Stommel et al. [119]	45	43	+	*T. gondii* seropositivity seen to be associated with cryptogenic epilepsy
	Ngô et al. [59]	149	–	+	*T. gondii* seropositivity seen to be significantly higher in epilepsy patients
	Abd El-Aal et al. [114]	178	36 ± 14	+	*T. gondii* seropositivity seen to be associated with epilepsy
	Eltantawy et al. [128]	192	9 ± 4	+	*T. gondii* seropositivity seen to be associated with cryptogenic epilepsy
	Allahdin et al. [129]	185	2 − 39	+	*T. gondii* seropositivity seen to be associated with epilepsy
	Eraky et al. [130]	90	5 ± 3	+	*T. gondii* seropositivity seen to be associated with cryptogenic epilepsy
	Zibaei et al. [131]	170	–	+	*T. gondii* seropositivity seen to be associated with epilepsy
	Yazar et al. [132]	150	36 ± 15	+	*T. gondii* seropositivity seen to be associated with epilepsy
	Cong et al. [74]	104	16 − 91	-	*T. gondii* seropositivity not seen to be associated with epilepsy
	Babaie et al. [118]	627	33 ± 10	-	*T. gondii* seropositivity not seen to be associated with epilepsy
	Alvarado-Esquivel et al. [125]	198	39 ± 16	-	*T. gondii* seropositivity not seen to increase the risk of certain types of epilepsy
	Akyol et al. [117]	150	28 ± 3.2	-	*T. gondii* seropositivity not seen to be associated with epilepsy
	Miman et al. [116]	55	7 − 16	-	*T. gondii* seropositivity not seen to be associated with epilepsy

Headaches	Koseoglu et al. [134]	104	33 ± 10	+	*T. gondii* seropositivity seen to be associated with migraines
	Flegr and Escudero [137]	1266	34 ± 12	+	*T. gondii* seropositivity seen to be associated with migraines
	Jouyani et al. [138]	100	20 − 60	+	*T. gondii* seropositivity seen to be associated with migraines
	Prandota [133]	108	10 − 66	-	*T. gondii* seropositivity not seen to be associated with migraines
	Alvarado-Esquivel et al. [136]	210	42 ± 15	-	*T. gondii* seropositivity not seen to be associated with headaches, but high serointensity seen to be associated with recurring headaches

OCD	Flegr and Horáček [140]	7471	32 − 35 ± 12	+	*T. gondii* seropositivity seen to be associated with OCD
	Miman et al. [16]	142	34 ± 12	+	*T. gondii* seropositivity seen to be significantly higher in OCD patients (48%) than controls (19%)
	Nayeri Chegeni et al. [143]	9873 (M)	All ages	+	*T. gondii* seropositivity seen to be associated with OCD
	Akaltun et al. [141]	60	Children and adolescents	+	*T. gondii* seropositivity seen to be associated with increased risk of OCD in children and adolescents
	Çakın Memik et al. [142]	87	12 ± 3	-	*T. gondii* seropositivity not seen to be associated with OCD
	Cong et al. [74]	82	16 − 91	-	*T. gondii* seropositivity not seen to be associated with OCD
	Zaki et al. [111]	179	35 ± 9	-	*T. gondii* seropositivity not seen to be associated with OCD
	Flegr and Escudero [137]	1256	34 ± 12	-	*T. gondii* seropositivity not seen to be associated with OCD

Sleep	Ahmad et al. [151]	2031	44 ± 17	-	*T. gondii* seropositivity not seen to be associated with sleep disturbances
	Corona et al. [153]	833	44.3 ± 17	-	*T. gondii* seropositivity not seen to be associated with sleep disturbances
	Dard et al. [152]	170	53 (median age)	-	*T. gondii* seropositivity not seen to be associated with sleep disturbances

Suicide	Okusaga et al. [49]	950	38 ± 11	+	*T. gondii* seropositivity seen to be associated with past suicidal behavior in young schizophrenic patients
	Yagmur et al. [154]	400	24 ± 7	+	*T. gondii* seropositivity seen to be associated with suicide
	Ling et al. [155]	WHO Europe^∗^	All ages	+	*T. gondii* seropositivity seen to be associated with suicide in women of postmenopausal age
	Bak et al. [156]	290	43 ± 16	+	*T. gondii* seropositivity seen to be associated with suicide attempts
	Coryell et al. [158]	222	35 ± 14	+	*T. gondii* seropositivity seen to be associated with suicide attempts
	Dickerson et al. [159]	72	40 ± 10	+	*T. gondii* seropositivity seen to be associated with suicide attempts
	Ansari-Lari et al. [160]	99	—	+	*T. gondii* seropositivity seen to be associated with suicide attempts
	Arling et al. [161]	257	40 ± 9.8	-	*T. gondii* seropositivity not seen to be associated with suicide
	Alvarado-Esquivel et al. [162]	156	34 ± 10	-	*T. gondii* seropositivity not seen to be associated with suicide attempts
	Sari and Kara [157]	100	16	-	*T. gondii* seropositivity not seen to be associated with suicide attempts
	Sugden et al. [202]	837	38	-	*T. gondii* seropositivity not seen to be associated with suicide attempts

Parkinson's disease	Miman et al. [23]	92	66 ± 12	+	*T. gondii* seropositivity seen to be associated with Parkinson's disease
	Ramezani et al.	150	63 ± 6	+	*T. gondii* seropositivity seen to be associated with Parkinson's disease
	Mahami-Oskouei et al. [163]	150	63 ± 11	-	*T. gondii* seropositivity not seen to be associated with Parkinson's disease
	Zhou et al. [164]	1086 (M)	62 ± 76	-	*T. gondii* seropositivity not seen to be associated with Parkinson's disease
	Celik et al. [48]	100	63 ± 12	-	*T. gondii* seropositivity not seen to be associated with Parkinson's disease
	Fallahi et al. [165]	230	75 ± 14	-	*T. gondii* seropositivity not seen to be associated with Parkinson's disease
	Alvarado-Esquivel et al. [203]	260	38 − 95	-	*T. gondii* seropositivity not seen to be associated with Parkinson's disease
	Gendy et al. [166]	90	53	-	*T. gondii* seropositivity not seen to be associated with Parkinson's disease

Schizophrenia	Torrey and Yolken [167]	(M)	–	+	*T. gondii* seropositivity seen to be associated with schizophrenia
	Leweke et al. [14]	148	30 ± 10	+	*T. gondii* seropositivity seen to be associated with recent onset schizophrenia
	Cetinkaya et al. [15]	200	37 ± 11	+	*T. gondii* seropositivity seen to be associated with schizophrenia
	Alipour et al. [179]	124	37 ± 10	+	*T. gondii* seropositivity seen to be associated with schizophrenia
	Muflikhah et al. [178]	94	–	+	*T. gondii* seropositivity seen to be associated with schizophrenia
	Burgdorf et al. [177]	81912	18 − 67	+	*T. gondii* seropositivity seen to be associated with schizophrenia
	Kezai et al. [180]	140	39 ± 9	+	*T. gondii* seropositivity seen to be associated with schizophrenia
	Fond et al. [181]	250	32 ± 8.6	+	*T. gondii* seropositivity seen to be associated with schizophrenia
	Cong et al. [74]	89	16 − 91	+	*T. gondii* seropositivity seen to be associated with schizophrenia
	Alvarado-Esquivel et al. [204]	218	43 ± 17	+	*T. gondii* seropositivity seen to be associated with schizophrenia
	Zaki et al. [111]	214	35 ± 9	+	*T. gondii* seropositivity seen to be associated with schizophrenia
	Ansari-Lari et al. [160]	251	18 − 59	+	*T. gondii* seropositivity seen to be associated with schizophrenia
	Wang et al. [205]	800	22 ± 5	+	*T. gondii* seropositivity seen to be associated with schizophrenia
	Khademvatan et al. [186]	100	36 ± 10	+	*T. gondii* seropositivity seen to be associated with schizophrenia, higher prevalence in females than males
	Flegr et al. [184]	173	35 ± 8	+	*T. gondii* seropositivity seen to be associated with schizophrenia, higher prevalence in males than females
	Al-Hussainy et al. [185]	177	–	+	*T. gondii* seropositivity seen to be associated with schizophrenia, higher prevalence in males than females
	Karabulut et al. [183]	145	41 ± 12	-	*T. gondii* seropositivity not seen to be associated with schizophrenia
	Xiao et al. [190]	837	–	+	*T. gondii* maternal seropositivity seen to be associated with schizophrenia in offspring
	Mortensen et al. [191]	1366	–	+	*T. gondii* maternal seropositivity seen to be associated with schizophrenia in offspring
	Brown [189]	186	25 ± 5	-	*T. gondii* maternal seropositivity not seen to be associated with schizophrenia in offspring

A linkage "+" indicates a statistically significant association with *T. gondii* as determined by the original authors, whereas a "-" indicates that the association does not meet their statistical threshold. ∗ represents data from the European Mortality Database and varies in countries and years. POMS: profile of mood states. (M): meta-analysis.
